# Exploring grandiose narcissism among surgeons: a comparative analysis

**DOI:** 10.1038/s41598-024-62241-6

**Published:** 2024-05-22

**Authors:** Henriette L. Moellmann, Majeed Rana, Monika Daseking, Hendric Petersohn, Madiha Rana

**Affiliations:** 1 University Hospital Duesseldorf, 40225 Duesseldorf, Germany; 2 Department of Psychology, University of Applied Sciences, 22143 Hamburg, Germany; 3 Department of Educational Psychology, Helmut-Schmidt-University/University of the Federal Armed Forces, 22043 Hamburg, Germany

**Keywords:** Narcissism, Admiration, Rivalry, Surgeons, Personality, Surgery, Psychology, Medical ethics

## Abstract

The concept of narcissism encompasses various personality traits, including cognitive, emotional and behavioural characteristics, which often lead to difficulties in maintaining a healthy self-esteem. This study examines the prevalence of narcissism traits (Admiration and Rivalry) in the surgical profession and their association with age, gender and professional experience. A total of 1390 participants (408 women, 982 men) took part in an online survey. The results show that female participants have significantly lower levels of rivalry than male colleagues. Additionally, age was found to be inversely correlated with both facets of narcissism, demonstrating that levels of narcissism decrease as age increases. Participants who are still in education tend to show higher levels of both facets. These results improve our understanding of personality traits in surgery and provide valuable insights for researchers and practitioners.

## Introduction

Narcissism is more common in surgery than in other disciplines^1^. However, there is little research on its prevalence and impact in surgeons, making it difficult to comprehensively assess its prevalence, particularly regarding gender differences. This personality trait exists along a continuum within personalities with varying degrees of expression^2–4^ Like other personality traits, narcissism, which is characterized by excessive self-admiration and the need for admiration, has a genetic component of around 50%^5^. Genetic predisposition and environmental factors such as upbringing and social environment interact to determine the expression of narcissism. The DSM-5 defines nine diagnostic criteria for pathological narcissism, including grandiosity, fantasies of success, lack of empathy and arrogance^6^. Wink^7^ has proposed two subtypes: vulnerability-sensitivity and grandiosity-exhibitionism, which are characterized by introversion and extraversion respectively. A comprehensive study by Back et al.^8^ from 2013 analyzed grandiose narcissism using the Narcissistic Admiration and Rivalry Concept (NARC), which divides narcissism into two subtypes and considers cognitive, affective-motivational and behavioral aspects. This is known as subclinical narcissism, which is to be distinguished from pathological narcissism and is the subject of this study. The admiration dimension includes the pursuit of uniqueness, grandiose fantasies, and charm, while the rivalry dimension includes the devaluation of others, the pursuit of superiority and aggression. These subtypes suggest that narcissism can also have functional aspects, which calls into question the assumption of inherent dysfunctionality.

Studies have shown that narcissism is more common in surgery than in other medical specialties, raising concerns about possible negative consequences^1,9^. Egocentric behaviors and disruptive attitudes pose a risk to surgical culture and patient safety as they can, for example, distract from patient care, increase the number of medical errors, and affect the well-being, turnover and cooperation of others in the perioperative environment^10–12^. In contrast, interpersonal effectiveness, and non-technical skills of surgeons (such as teamwork, communication, and co-operation) can improve surgical performance^13^ of the individual and the whole team^14^, requiring a shift towards a more positive, humanistic medical culture^14^. The conceptualization of 'surgeon ego' has highlighted various manifestations such as narcissism, arrogance and dominance that impact on patient outcomes and the perioperative environment^9–11,15^.

While the influence of gender on the "surgeon ego" remains unclear, disruptive behaviors are mainly attributed to male colleagues, as men have higher levels of narcissism in the general population^16^. It is therefore of particular interest to further investigate possible gender differences in the functional and dysfunctional aspects of narcissism in surgeons. This research could provide important insights into the dynamics of the profession and contribute to the development of personalized training programs to improve the working environment and optimize patient care. In addition, research could be conducted to analyze the possible dependence of narcissism levels on the specialties within surgery. This could help to identify specific areas where action should be taken to promote a healthy working environment and minimize negative effects on patient care. Moreover, examining the role of age could contribute to a more comprehensive understanding of how narcissism manifests in this professional group and its implications for patient care and the work environment.

The aim of this study is therefore to assess the expression of narcissistic personality traits in surgeons and to investigate relationships with factors such as gender, age and specialty, as these may influence the working environment, patient care, perceptions of gender and career choice.

## Material and methods

This investigation was approved by the local ethics committee of the University of Applied Sciences in Hamburg (EKEFH01/22). It is confirmed that all research was conducted in accordance with relevant guidelines and regulations, and all participants provided informed consent by participating in the online survey. The research adhered to the principles outlined in the Declaration of Helsinki. Approximately 11,400 emails containing the link to the questionnaire were dispatched via surgical societies and hospital registries. Out of a total of 1427 participants, responses on socio-demographic data and the Narcissistic Admiration and Rivalry Questionnaire (NARQ) from 1390 doctors were evaluated. The study was carried out using Questback (https://www.questback.com/). Initially, participants were given a brief introduction, and they were adequately informed about data privacy protection. In the first part of the survey, participants were asked to provide socio-demographic data such as age, gender, and educational as well as medical discipline. Subsequently, the assessment for the NARQ was captured. The online survey concluded with a debriefing. Before utilizing the responses, the data were manually screened for anomalies and suspicious responses (i.e., very short processing times and similar or identical answers), but it was not necessary to exclude any participants.

The NARQ was used to evaluate narcissism. This questionnaire is a test procedure to assess the grandiose narcissistic type in two differentiated subtypes (Back et al. 2013) and has successfully been conducted to validate narcissism^20–22^. NARQ consists out of 2 Likert scales: Narcissistic Admiration and Narcissistic Rivalry. Each scale is composed of 3 facets with 3 items. The three facets of admiration are: (1) grandiosity, (2) striving for uniqueness and (3) charm. The facets for rivalry are: (1) Devaluation, (2) Striving for supremacy and (3) Aggressiveness. Participants can agree or disagree choosing on a scale between "1 = not agree at all" to "6 = completely agree"^20,21^. Cronbach’s Alpha for the two scales show satisfying results with 0.87 (*admiration*) and 0.83 (*rivalry*).

- To what extent can narcissistic traits be found among surgeons?
- What are the differences between women and men regarding narcissistic traits?
- What is the relationship between age and education and narcissistic traits?

### Statistical analysis

The determined values of the measurements as well as the clinical data were statistically analyzed using jamovi (2.2.5). Mean differences are tested with independent T-Test (t) when significant outliers, identified with boxplots were excluded, normal distribution of the dependent variable, tested with Shapiro–Wilk-Test and homoscedasticity, tested with Levene’s test, were met. Mean Differences of non-normal dependent variable data is analyzed with Mann–Whitney-U test (U). One-way ANOVA was used to compare means across groups. For the correlation between two parameters, the Pearson product-moment correlation (r) is calculated if the assumptions of linear relationship and normal distribution of the data (assessed with the Shapiro–Wilk test) have been met. For non-normal data Spearman's rank-order correlation (ρ) is calculated. A p-value of < 0.05 was defined as significant, a value of < 0.01 as very significant, and a value of < 0.001 as highly significant. A significance level of p > 0.05 is set for hypothesis testing.

### Institutional review board statement

Ethicl approval was waived by the local Ethics Committee of the University of Applied Science in Hamburg (EKEFH01/22).

### Informed consent

Informed consent was obtained from all individual participants included in the study.

## Results

In this investigation the 408 female participants show NARQ_admiration-values between 9.00 and 52.00 (M = 23.00, SD = 7.61), the male participants (n = 982) between 9.00 and 54.00 (M = 24.00, SD = 7.75). The NARQ_rivalry-values for female range between 9.00 and 46.00 (M = 15.20, SD = 5.35), for male between 9.00 and 54.00 (M = 16.00, SD = 5.79). The values in NARQ_admiration and also NARQ_rivalry are not normally distributed, as the Shapiro–Wilk test revealed (α = 0.05). With p = 0.87 or 0.18, there is a homogeneity of variance according to Levene's test. Details on the descriptive date are presented in Table 1.

**Table 1:** Descriptive statistics.

	Total	Male	Female
Age			
Years (Mean ± SD)	43 ± 12.6	40.8 ± 11.8	39.2 ± 11.3
n	1390	982 (70.6%)	408 (29.4%)
Surgical speciality			
Completed: yes	1005 (72.3%)	794 (80.9%)	211 (51.7%)
no	381 (27.4%)	186 (18.9%)	195 (47.8%)
NA	4 (0.3%)	2 (0.2%)	2 (0.5%)
General and Visceral Surgery	105 (7.6%)	65 (61.9%)	40 (38.1%)
Surgery	624 (44.9%)		
Vascular surgery	38 (2.7%)	26 (68.4%)	12 (31.6%)
Cardiac surgery	57 (4.1%)	42 (73.7%)	15 (26.3%)
Paediatric surgery	30 (2.2%)	14 (46.7%)	16 (53.3%)
Oral and maxillofacial surgery	34 (2. 4%)	24 (70.6%)	10 (29.4%)
Neurosurgery/spinal surgery	107 (7.7%)	74 (69.2%)	33 (30.8%)
Plastic surgery/hand surgery	27 (1.9%)	16 (59.3%)	11 (40.7%)
Thoracic surgery	18 (1.3%)	15 (83.3%)	3 (16.7%)
Trauma surgery/orthopaedics	350 (25.2%)	282 (80.6%)	68 (19.4%)

Overview of demographic data and qualifications in the overall collective and for women and men (Age, Surgical speciality).

**Figure 1:**
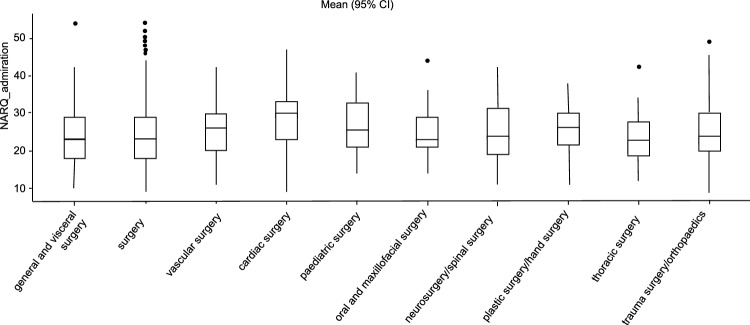
Comparison admiration and gender. Average difference between the two gender groups in relation to the NARQ_adminiration values.

**Figure 2:**
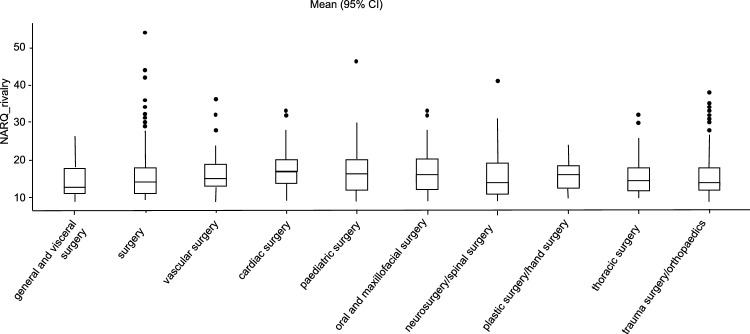
Comparison of rivalry and gender. Average difference between the two gender groups in relation to the NARQ_adminiration values.

The conducted Mann–Whitney U test showed a significantly lower value in NARQ_rivalry for the female participants with a small effect size compared to the men (U = 184,501, p = 0.02, r_rb_ = 0.08). There was no significance regarding the NARQ_admiration (U = 187,112, p = 0.052, r_rb_ = 0.07) between the female and male participants (see Figs. 1 and 2).

Regarding the specialty, significant differences are present for admiration (F(9,153) = 2.65, p = 0.005) and rivalry (F(9,152) = 2.21, p = 0.037). An overview of the values, depending on the specialty, is shown in Table 2.

**Table 2:** Overview of NARQ_admiration and NARQ_rivalry values subdivided according to specialist disciplines.

	NARQ_admiration				95% CI		NARQ_rivalry				95% CI	
	n	Mean	SD	SE	Mean lower bound	Mean upper bound	n	Mean	SD	SE	mean lower bound	mean upper bound
General and visceral surgery	105	23.3	7.5	0.736	21.9	24.7	105	14.4	4.3	0.423	13.6	15.2
Vascular surgery	38	25.4	7.5	1.21	23	27.7	38	16.3	5.8	0.946	14.4	18.1
Cardiac surgery	57	28.2	8.7	1.15	26	30.5	57	17.6	5.4	0.714	16.2	19
Pediatric surgery	30	26.3	7.7	1.4	23.6	29	30	17.3	7.7	1.41	14.6	20.1
Oral and maxillofacial surgery	34	25.1	6.7	1.15	22.9	27.4	34	17.3	6.4	1.1	15.2	19.5
Neurosurgery/spinal surgery	107	24.9	7.7	0.742	23.5	26.4	107	15.8	5.8	0.559	14.7	16.9
Plastic surgery/hand surgery	27	26.1	6.2	1.2	23.8	28.5	27	16	4.4	0.852	14.3	17.7
Thoracic surgery	18	24.3	7.9	1.85	20.6	27.9	18	16.7	6.8	1.6	13.5	19.8
Trauma surgery/orthopedics	350	24.8	7.2	0.384	24.1	25.6	350	15.6	5	0.267	15.1	16.1

**Figure 3:**
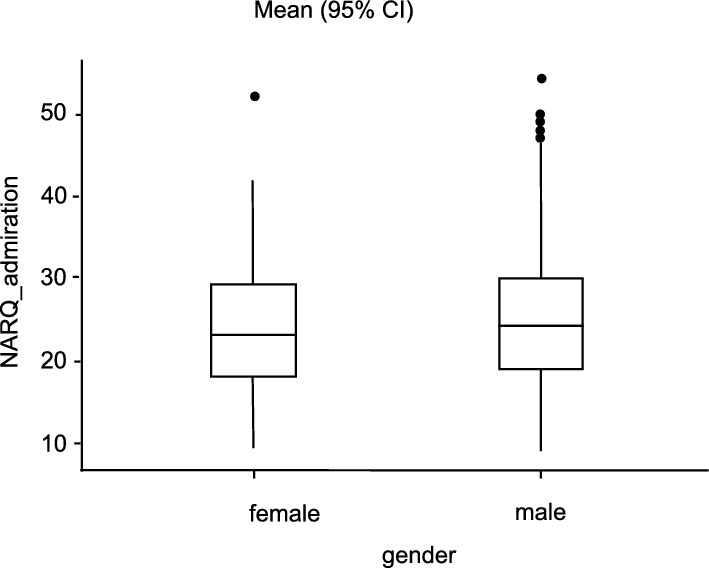
Comparison of the specialty disciplines. Average difference between the specialties in relation to the NARQ_adminiration values (General and Visceral Surgery, Vascular Surgery, Cardiac Surgery, Pediatric Surgery, Oral and Maxillofacial Surgery, Neurosurgery/spinal surgery, Plastic surgery/hand surgery, Thoracic surgery and Trauma surgery/orthopedics).

With p = 0.002, the general and visceral surgeons showed significantly lower values in NARQ_admiration than the cardiac surgeons. The comparison of the examined disciplines did not reveal any significant differences (see Fig. 3).

**Figure 4:**
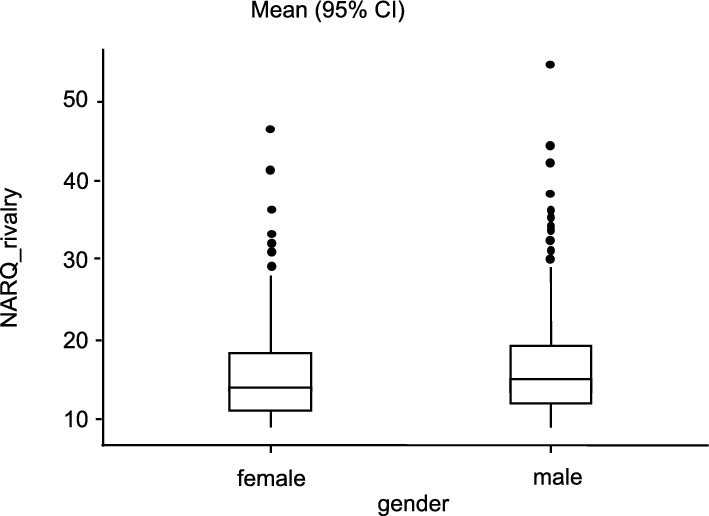
Comparison of the specialty disciplines. Average difference between the specialties in relation to the NARQ_rivalry values (General and Visceral Surgery, Vascular Surgery, Cardiac Surgery, Pediatric Surgery, Oral and Maxillofacial Surgery, Neurosurgery/spinal surgery, Plastic surgery/hand surgery, Thoracic surgery and Trauma surgery/orthopedics).

When evaluating the NARQ_rivalry values, cardiac surgeons show significantly higher values (p = 0.010) than general and visceral surgeons. The comparison of the different specialties indicates that there are no significant differences (see. Figure 4).

**Figure 5:**
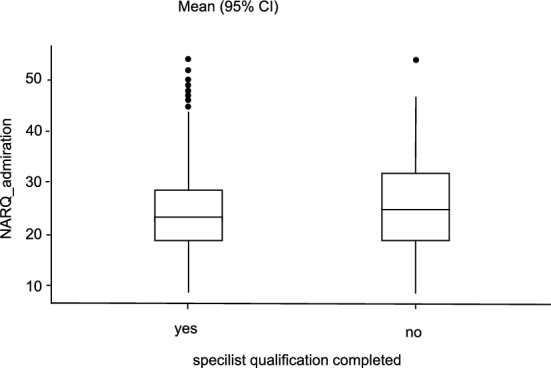
Comparison of completed specialist qualification. Average difference of NARQ_admiration values in relation to completed specialist training.

**Figure 6:**
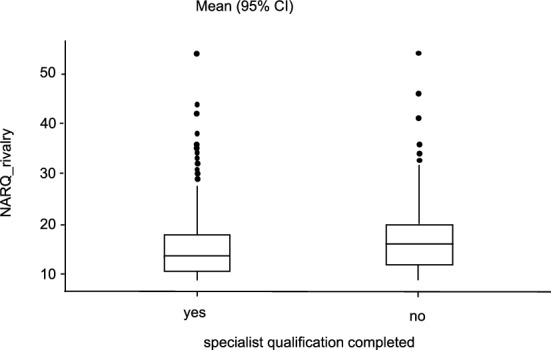
Comparison of completed specialist qualification. Average difference of NARQ_rivalry values in relation to completed specialist training.

Looking at the relationship between age and scores on the two narcissism dimensions, it is noticeable that a significant correlation with r = − 0.114, p < 0.001 and r = − 0.183, p < 0.001 could be shown for both NARQ_admiration and NARQ_rivalry (see Figs. 5 and 6). Older participants tend to show significantly lower values. It is important to interpret these results with caution, as the significant correlations observed may be confounded by the concurrent increase in qualifications with age, which could potentially influence these findings. This is also reflected in the completed specialist qualification. Here, the participants who have not yet completed their specialist show higher values (NARQ_admiration: U = 172,140; p = 0.004; NARQ_rivalry: U = 160,590; p < 0.001). Comparing the individual disciplines as a function of gender, only thoracic surgery (U = 5.50, p = 0.05) showed significantly higher values in NARQ_rivalry in men.

## Discussion

The proportion of female medical students has risen sharply (up to 60%), but few opt for surgical training or a career aiming for a surgical leadership position^23^. This may be since many "typically male" traits, as described in this study in relation to narcissism, discourage women from entering surgical careers^17^. In addition to reasons such as perceived arrogance and intimidation, other reasons such as pressure to perform are also described^18^. However, given that studies have demonstrated that patients of female surgeons tend to have better outcomes compared to those of male surgeons, it is imperative to enhance the appeal of this profession to women, underscoring the potential for significant improvements in surgical outcomes^17,19^. In the present study, we found lower values for NARQ_rivalry in women compared to men. This is consistent with the findings of Grijalva et al. (2015), who showed that men are more likely to exploit others and believe in their exceptionality to gain advantages^16^. Weidmann et al. also showed higher values for men in their meta-analysis^24^. Women therefore show fewer socially incompatible characteristics when interacting with others, which would underpin the success of women in this profession, but at the same time also provides an indication of why they are unwilling or unable to compete with their male colleagues.

Whilst this is just one of many reasons why it can be difficult for women to gain a foothold in surgery, it would be important in this context to address a possible perception of arrogance and intimidation in surgery and to create a supportive working environment. Further research should be conducted to identify the specific barriers for women in surgery and develop targeted solutions. By comprehensively analyzing gender dynamics, effective strategies can be developed to promote gender equality in this field.

Comparing medical disciplines, cardiac surgeons show higher values for both dimensions, orthopedic surgeons rank in the midfield, and general surgeons show the lowest values. A similar distribution was shown by Bucknall et al. who applied the NPI among British healthcare professionals^1^. Furthermore, it can be assumed that people with higher narcissism values choose disciplines that are associated with higher prestige. That is why these people are mainly found in surgery and they are in cardiac surgery. It can be assumed that a reasonable degree of self-confidence is required in surgical specialties. This allows to make difficult decisions as well as to act quickly and prudently. However, this can also lead to difficult interaction and ultimately have a negative impact on patients. It may therefore be necessary to strive for more action and teamwork, especially in these disciplines.

Furthermore, we found that older physicians show significantly lower scores in NARQ_admiration as well as in NARQ_rivalry. Yet, prevailing research on narcissism is mostly concerned with young adults knowing that there are differentiations of narcissism with changing ages. In 2009, Foster et al. demonstrated in a large-scale cross-sectional study that scores in the NPI decline steadily from age 15–54 before rising again from age 55^25^. This is in line with our results. In their cross-sectional analyses with a total sample of 270,029 participants, Weidmann et al. showed that there are consistent linear age effects, with narcissism being most prevalent in young adults^24^. The observed decrease in narcissism scores with age may also be influenced by higher qualifications typically attained by older individuals in our sample. Given the dataset limitations, it was not feasible to fully disentangle the effects of age from those of increasing professional qualifications.

Regarding the highest academic degree attained, no significant differences emerge. Leadership positions are frequently sought by narcissists. Simultaneously, narcissists tend to act in their own self-interest while compromising the needs and interests of others. Campbell et al. described this behavioral pattern as the two sides of narcissism: a light side, and a dark side^26^. Besides leadership aspirations, in this study, younger colleagues who have not yet completed their education show higher scores in admiration and rivalry. These results underline the importance of a differentiated approach to the study of narcissism and its effects on the behavior and performance of physicians.

When interpreting the results of this study, we acknowledge that healthcare organizations have changed and with them the demands on physicians’ leadership styles^27,28^. Healthcare nowadays assumes hybrid role joining clinical and operative activities^29^. In addition to professional competence, additional attributes such as technical knowledge and expertise are of immense relevance^30,31^. When examining attributes crucial for leadership success, it's important to recognize that personality traits like narcissism can have a dual impact. On one hand, narcissism may enhance personal assertiveness and decisiveness, contributing positively to perceived leadership capabilities. On the other hand, these same traits can lead to poorer team performance and reduced overall achievement due to potential interpersonal conflicts and self-serving behaviors^32–34^. Healthcare organizations should therefore adopt a strategic approach to leadership development, balancing technical expertise with management skills to ensure optimal outcomes^35^.

## Conclusion

In this study, we have shed light on personality traits in surgery. First, we found that narcissistic personality dimensions are present in surgical specialties. Our analysis revealed significant differences in the levels of admiration and rivalry based on age and gender. Specifically, these traits vary significantly across different age groups and between genders, reflecting distinct patterns in how these demographics manifest narcissistic traits. Second, female participants scored significantly lower on rivalry than male participants. Thirdly, our findings indicate that participants who have not yet completed their professional training exhibit higher scores in both admiration and rivalry. This suggests that earlier stages of professional development might be associated with higher levels of narcissistic traits, which appear to decrease as individuals gain more experience and complete their training.

The study has several limitations, including sampling bias due to the regionally limited sample, self-report measures susceptible to social desirability bias, a cross-sectional design that precludes causal inference, and limited variables such as gender and age. Limited cultural contextualization is also a constraint. Future research should consider these limitations to enhance the validity and generalizability of the findings. Overall, this research underscores the relevance of studying narcissism in surgery and its implications for both practitioners and healthcare organizations in fostering positive workplace environments and patient outcomes.

## Data Availability

The data presented in this study are available on request from the corresponding author. The data are not publicly available due to privacy regulations.

## Abbreviations

NARC: Narcissistic Admiration and Rivalry Concept
NARQ: Narcissistic Admiration and Rivalry Questionnaire
NHS: UK National Health Service
NPI: Narcissistic Personality Inventory
MLCF: Medical Leadership Competency Framework

## References

[1] Bucknall V, Burwaiss S, MacDonald D, Charles K, Clement R (2015). Mirror mirror on the ward, who's the most narcissistic of them all? Pathologic personality traits in health care. CMAJ.

[2] Neumann E (2010). Offener und verdeckter Narzissmus. Psychotherapeut.

[3] Sachse, R., Sachse, M. & Fasbender, J. *Klärungsorientierte Psychotherapie der narzisstischen Persönlichkeitsstörung* (Hogrefe, 2011).

[4] Vater A, Roepke S, Ritter K, Lammers C-H (2013). Narzisstische Persönlichkeitsstörung. Psychotherapeut.

[5] Polderman TJC (2015). Meta-analysis of the heritability of human traits based on fifty years of twin studies. Nat. Genet..

[6] Association AP (2013). Diagnostic and Statistical Manual of Mental Disorders. DSM-5.

[7] Wink P (1991). Two faces of narcissism. J. Pers. Soc. Psychol..

[8] Back MD (2013). Narcissistic admiration and rivalry: Disentangling the bright and dark sides of narcissism. J. Pers. Soc. Psychol..

[9] Myers CG, Lu-Myers Y, Ghaferi AA (2018). Excising the "surgeon ego" to accelerate progress in the culture of surgery. BMJ.

[10] Rosenstein AH, O'Daniel M (2005). Disruptive behavior and clinical outcomes: Perceptions of nurses and physicians. AJN.

[11] Rosenstein AH, O'Daniel M (2006). Impact and implications of disruptive behavior in the perioperative arena. J. Am. Coll. Surg..

[12] Cochran A, Elder WB (2015). Effects of disruptive surgeon behavior in the operating room. Am. J. Surg..

[13] Hull L (2012). The impact of nontechnical skills on technical performance in surgery: A systematic review. J. Am. Coll. Surg..

[14] Sakran JV (2013). Changing the surgical culture, one apple at a time. Bull. Am. Coll. Surg..

[15] Shapiro J (2018). Confronting unprofessional behaviour in medicine. BMJ.

[16] Grijalva E (2015). Gender differences in narcissism: A meta-analytic review. Psychol. Bull..

[17] Wallis CJ (2017). Comparison of postoperative outcomes among patients treated by male and female surgeons: A population based matched cohort study. The BMJ.

[18] Hill EJR, Bowman KA, Stalmeijer RE, Solomon Y, Dornan T (2014). Can I cut it? Medical students' perceptions of surgeons and surgical careers. Am. J. Surg..

[19] Logghe H, Jones C, McCoubrey A, Fitzgerald E (2017). #ILookLikeASurgeon: Embracing diversity to improve patient outcomes. The BMJ.

[20] Grove JL, Smith TW, Girard JM, Wright AG (2019). Narcissistic admiration and rivalry: An interpersonal approach to construct validation. J. Pers. Disord..

[21] Leckelt M (2018). Validation of the Narcissistic Admiration and Rivalry Questionnaire Short Scale (NARQ-S) in convenience and representative samples. Psychol. Assess..

[22] Grosz MP (2019). A comparison of unidimensionality and measurement precision of the narcissistic personality inventory and the narcissistic admiration and rivalry questionnaire. Assessment.

[23] Ganschow P (2012). Einstellung von Studierenden zu einer chirurgischen Karriere - ein globales Phänomen?. Zentralblatt Chir..

[24] Weidmann R (2023). Age and gender differences in narcissism: A comprehensive study across eight measures and over 250,000 participants. J. Pers. Soc. Psychol..

[25] Foster JD, Misra TA, Reidy DE (2009). Narcissists are approach-oriented toward their money and their friends. J. Res. Pers..

[26] Campbell WK, Hoffman BJ, Campbell SM, Marchisio G (2010). Narcissism in organizational contexts. Hum. Resour. Manag. Rev..

[27] Lega F, Prenestini A, Spurgeon P (2013). Is management essential to improving the performance and sustainability of health care systems and organizations? A systematic review and a roadmap for future studies. Value Health.

[28] Kirkpatrick I, Bullinger B, Lega F, Dent M (2013). The translation of hospital management models in European health systems: A framework for comparison. Br. J. Manag..

[29] Llewellyn S (2001). `Two-Way Windows': Clinicians as medical managers. Organization Stud..

[30] Braithwaite J, Westbrook MT (2004). A survey of staff attitudes and comparative managerial and non-managerial views in a clinical directorate. Health Serv. Manag. Res..

[31] Fulop L (2012). Leadership, clinician managers and a thing called "hybridity". J. Health Org. Manag..

[32] Chan KY, Drasgow F (2001). Toward a theory of individual differences and leadership: Understanding the motivation to lead. J. Appl. Psychol..

[33] Mascia D, Dello Russo S, Morandi F (2015). Exploring professionals' motivation to lead: A cross-level study in the healthcare sector. Int. J. Hum. Resour. Manag..

[34] Judge TA, Rodell JB, Klinger RL, Simon LS, Crawford ER (2013). Hierarchical representations of the five-factor model of personality in predicting job performance: Integrating three organizing frameworks with two theoretical perspectives. J. Appl. Psychol..

[35] Di Vincenzo F, Angelozzi D, Morandi F (2021). The microfoundations of physicians' managerial attitude. BMC Health Serv. Res..

